# Transcriptome reprogramming by cancer exosomes: identification of novel molecular targets in matrix and immune modulation

**DOI:** 10.1186/s12943-018-0846-5

**Published:** 2018-07-16

**Authors:** Fatima Qadir, Mohammad Arshad Aziz, Chrisdina Puspita Sari, Hong Ma, Haiyan Dai, Xun Wang, Dhiresh Raithatha, Lucas Girotto Lagreca Da Silva, Muhammad Hussain, Seyedeh P. Poorkasreiy, Iain L. Hutchison, Ahmad Waseem, Muy-Teck Teh

**Affiliations:** 1 Centre for Oral Immunobiology & Regenerative Medicine, Institute of Dentistry, Barts & The London School of Medicine and Dentistry, Queen Mary University of London, The Blizard Building, 4, Newark Street, E1 2AT, London, England UK; 2 Department of Oral & Maxillofacial Surgery, China-British Joint Molecular Head and Neck Cancer Research Laboratory, Affiliated Hospital & School of Stomatology, Guizhou Medical University, Guizhou, China; 3 Department of Oral & Maxillofacial Surgery, Barts & The London NHS Trust, London, England UK; 4 Cancer Research Institute, Affiliated Cancer Hospital & Institute of Guangzhou Medical University, Guangzhou, China

**Keywords:** FOXM1, CEP55, ESCRT, exosomes, Extracellular vesicles, Reprogramming, Biomarkers

## Abstract

**Background:**

Exosomes are extracellular vesicles released by almost all cell types, including cancer cells, into bodily fluids such as saliva, plasma, breast milk, semen, urine, cerebrospinal fluid, amniotic fluid, synovial fluid and sputum. Their key function being intercellular communication with both neighbouring as well as distant cells. Cancer exosomes have been shown to regulate organ-specific metastasis. However, little is known about the functional differences and molecular consequences of normal cells responding to exosomes derived from normal cells compared to those derived from cancer cells.

**Methods:**

Here, we characterised and compared the transcriptome profiles of primary human normal oral keratinocytes (HNOK) in response to exosomes isolated from either primary HNOK or head and neck squamous cell carcinoma (HNSCC) cell lines.

**Results:**

In recipient HNOK cells, we found that regardless of normal or cancer derived, exosomes altered molecular programmes involved in matrix modulation (MMP9), cytoskeletal remodelling (TUBB6, FEZ1, CCT6A), viral/dsRNA-induced interferon (OAS1, IFI6), anti-inflammatory (TSC22D3), deubiquitin (OTUD1), lipid metabolism and membrane trafficking (BBOX1, LRP11, RAB6A). Interestingly, cancer exosomes, but not normal exosomes, modulated expression of matrix remodelling (EFEMP1, DDK3, SPARC), cell cycle (EEF2K), membrane remodelling (LAMP2, SRPX), differentiation (SPRR2E), apoptosis (CTSC), transcription/translation (KLF6, PUS7). We have also identified CEP55 as a potential cancer exosomal marker.

**Conclusions:**

In conclusion, both normal and cancer exosomes modulated unique gene expression pathways in normal recipient cells. Cancer cells may exploit exosomes to confer transcriptome reprogramming that leads to cancer-associated pathologies such as angiogenesis, immune evasion/modulation, cell fate alteration and metastasis. Molecular pathways and biomarkers identified in this study may be clinically exploitable for developing novel liquid-biopsy based diagnostics and immunotherapies.

**Electronic supplementary material:**

The online version of this article (10.1186/s12943-018-0846-5) contains supplementary material, which is available to authorized users.

## Background

Exosomes are extracellular nano-sized (< 150 nm) membrane vesicles released by almost all cell types, including cancer cells, into almost all bodily fluids. They are spherical bilayered proteolipids harbouring specific proteins [1], RNA [2] and DNA [3]. Non-coding RNA (microRNA, siRNA and piRNA) and mRNA are key cargos of exosomes [2]. Their key function being intercellular communication with both neighbouring as well as distant cells [2]. It has been suggested that tumour cells exploit this intercellular communication mechanism to confer target cell reprogramming that leads to cancer-associated pathologies such as angiogenesis, immune evasion/modulation, cell fate alteration and metastasis. Emerging evidence suggests that tumour viruses also exploit the exosomal message delivery system to induce pathogenesis. Identification of oncogenic exosomal RNA is prerequisite to the understanding of tumour pathophysiology.

Protein composition of exosomes is informative of any existing pathology as they can carry tumour antigens and inflammatory mediators. They also carry customary proteins including HSC70, TSG101 and tetraspanins [1], in addition they carry specific proteins which are involved in vesicle formation and trafficking such as ALIX (Apoptosis linked gene 2-interacting protein X) [4]. Exosomes are enriched in tetraspanins, a family of proteins that organizes membrane microdomains called tertraspanin enriched microdomains, by forming clusters and interacting with transmembrane and cytosolic signalling proteins [5]. Among tetraspanin CD9, CD63, CD81, CD82 and CD151 have a broad tissue distribution. They are involved in biological processes including cell adhesion, motility, membrane fusion, signalling and protein trafficking [6].

Biogenesis of intraluminal vesicle (ILV, which later become exosomes when excreted) involves endosomal sorting complex required for transport (ESCRT). ESCRT consist of approximately 20 proteins that assemble into four complexes ESCRT-0, I, II and III with associated proteins VPS4 (Vacuolar protein sorting- associated protein 4), VTA1 (vesicle trafficking 1) and ALIX forming ESCRT accessory complex [7]. ESCRT-0 complex recognizes and segregates ubiquitylated proteins in endosomal membrane. ESCRT I and II deform the membrane into buds with sequestered cargo. ESCRT III is responsible for cleavage into free vesicles [8]. The mechanism by which ESCRT III complex detaches ILV into multi-vesicular body is similar to final cut between two dividing daughter cells [9]. Recent studies have shown formation of a helix with a centrosomal protein (CEP55), which translocates to the mid-body during the late phase of cell division and functions as a scaffold for components of the abscission machinery. CEP55 interacts with ESCRT and ALIX-binding region (EABR) [10]. Previously we have shown that CEP55 is a downstream target of FOXM1, an oncogene that regulates cell cycle, DNA repair and maintenance of genomic stability [11, 12]. This study investigated the presence of CEP55 protein in normal and cancer exosomes.

The presence of exosomes in bodily fluids (eg., saliva) represents a promising surrogate approach to investigate tumour exosomal RNA biomarkers which has important clinical implications for developing non-invasive salivary diagnostics and therapeutics [13]. Human saliva is an ideal fluid for developing non-invasive diagnostics and salivary biomarkers have been demonstrated in clinical studies showing promising diagnostic potentials but lacking in sensitivity mostly due to complexity of saliva [13]. Hence, the ability to purify the highly stable (RNA cargo within exosomes are resistant to RNase [2]) and protected biomolecules within exosomes helps in reducing background noise in a highly complex and heterogeneous environment such as saliva [13]. Most of the salivary exosome studies to date have been restricted to characterization of normal healthy samples [13]. Emerging studies began looking at biochemical properties of disease-derived saliva exosomes but most of these studies focused on proteomics analysis [1, 13].

HNSCC is diagnosed in over half a million individuals worldwide each year, with an expected global incidence of 750,000 by 2015 [14]. According to the 8th national annual head and neck cancer audit report published by UK Health and Social Care Information Centre, there were 8272 cases within England and Wales in 2012. Survival rates are poor (10–30% at 5 years) among patients presenting with advanced disease. Early detection of precancer lesions coupled with early intervention could significantly improve patient outcome, reduce mortality and alleviate healthcare costs. Unfortunately, conventional histopathology is currently unable to accurately identify which individual lesions from the oral potentially malignant disorders spectrum will transform to squamous cell carcinoma (SCC). Given similar pathogenesis of other epithelial SCCs, the same clinical dilemmas apply to the management of vulva and skin premalignancies. Current screening methods for HNSCC in otherwise symptom-free persons include the use of oral cytology (brush biopsy), toludine blue staining and various light-based detection systems. More advanced screening methods such as salivary proteomics and antibody-based detection are under investigation. However, the effectiveness of these oral screening adjuncts in detecting early cancer remains unproven [15]. Hence, there is an urgent clinical need to explore novel cancer biomarkers with better sensitivity and specificity. Salivary exosomal RNA represents a promising new avenue for developing a non-invasive HNSCC screening tool [13]. In an attempt to identify novel biomarkers for early detection of HNSCC, this study characterised and investigated normal and cancer-derived exosomes and their transcriptome modulating profiles on recipient primary human normal oral keratinocyte cells.

## Methods

### Cell culture

Normal primary human oral keratinocytes were cultured in serum free medium (SFM) containing 15 ng/ml of human recombinant epidermal growth factor cat no. 10450–013), 62.5 μg/ml bovine pituitary extract (cat no. 13028–014) and 1% penicillin/streptomycin (cat no. 15070–063 from Life technologies UK). HNSCC and transformed cell lines were cultured in Dulbecco’s Modified Eagle Medium (DMEM) with 10% foetal bovine serum (FBS) (cat no. 02–00-850, from First Link Ltd. UK) and 1% penicillin/streptomycin. All cells were cultured in a humidified incubator with 5% CO2/95% atmospheric air at 37 °C. Due to the abundance of bovine exosomes in FBS, we have found that human exosomes, although still detectable (by qPCR against specific human markers), were partially masked by the much larger quantity of bovine exosomes (data not shown). Hence, for cells grown in FBS-containing DMEM, to prevent contamination from bovine exosomes, once cells reached ~ 90% confluent (~ 2 × 10^7^ cells in a 175 cm^2^ flask), we switched to culturing cells in SFM. All cells were left to grow in SFM for 3 days prior to exosome isolation. Additional information regarding each cell line used in this study can be found in (Additional file 1).

### Isolation of exosomes by ultracentrifugation

Exosomes were isolated from cell culture supernatant according to well-established ultracentrifugation method [5, 17, 18] with minor modification. Briefly, the conditioned SFM supernatant was collected and centrifuged in 50 mL tubes at the speed of 500×g for 10 min to remove cellular debris and apoptotic bodies. The supernatant was then centrifuged (SORVALL® Discovery™ SE ultracentrifuge with a SORVALL® T-865 Fixed 23.5° Angle Rotor, k-factor = 51.7) at the speed of 16,500×g for 20 mins to collect microvesicles. Special polycarbonate high speed centrifuge tubes from Thermo Scientific (cat. no. 314348) with screw cap lids (cat no. 314347) were used for high speed ultracentrifugation. The supernatant was filtered through a 0.22 μm filter to remove protein and debris prior to ultracentrifugation at 118,000×g for 70 mins to pellet exosomes. The last ultracentrifugation step was repeated to wash exosomes pellet in PBS.

### Scanning electron microscopy

Exosome pellet was re-suspended and fixed in PBS containing 2.5% glutaraldehyde for 1 h at room temperature. Fixed exosomes were washed by adding 20 mL PBS followed by ultracentrifugation at 118000×g for 70 mins. The exosome pallet was resuspended in 100 μl of PBS and incubated on fibronectin-precoated 13 mm round coverslips, without allowing the coverslips to dry, the samples were incubated overnight at 37 °C. Coverslips were then dehydrated in ascending series of ethanol concentrations from 30, 50, 70, 80, 90 to 95% for 5 mins each, followed by two 5 mins incubations in 100% ethanol. The samples were then chemically dried in 100% HMDS (Hexamethyldisilazane) for 3 mins and allowed to air dry at 37 °C for 30 mins. Double-sided adhesive carbon coated conductive discs were used to secure the coverslips onto the SEM stubs. A carbon conducting cement was used to aid a conducting pathway between the stub and the coverslips. The cement was allowed to dry for 24 h prior to coating with gold or carbon particles. Scanning electron micrographs (at 0.5 to 30 kV) were obtained using an FEI Inspect F system. The xT microscope control software was used to control the operation of the microscope while the image capturing software was used to obtain images. SEM was supervised by Dr. Russell Bailey at the Nano Vision centre, QMUL.

### Transmission electron microscopy

Exosome pellet was resuspended and fixed in 100 μl of 4% (*w*/*v*) paraformaldehyde for 10 mins and 5 μl of the fixed exosomes were placed on carbon coated EM grids (Catalogue no. S160–4 carbon film, 400 Mesh Cu by Agar scientific) for 20 mins. A drop of 100 μl of PBS on parafilm sheet was used to wash the grid (membrane side down). The grids were transferred to a 50 μl drop of 2.5% (w/v) gluteraldehyde for 10 mins, followed by eight washes in 100 μl of molecular-grade DNase/RNase/Protease-free water (W4502, Sigma-Aldrich) [16]. The samples were stained by 0.4% (w/v) lead citrate for 1 min. Stained grids were washed twenty times with distilled water and air dried on filter paper. TEM was supervised by Dr. Russell Bailey from Nano Vision centre QMUL.

### Immuno-gold TEM

Exosomes were fixed in 100 μl of 4% (w/v) paraformaldehyde from which 5 μl was placed on carbon coated electron microscopy grids. The grids were covered and left for 20 mins to facilitate adsorption. Further, the grids were washed in 100 μL drops of PBS and transferred in PBS/50 mM ammonium chloride (NH_4_CL) for 3 mins. The grids were transferred to blocking buffer (10% foetal calf serum) for 10 mins followed by a transfer to 5 μl drops of CEP55 antibody in the dilution of 1:50 in blocking buffer for 30 mins. The grids were washed multiple times in washing buffer for 3 mins. The grids were incubated with secondary (bridging) antibody diluted in blocking buffer for 30 mins and transferred to 100 μl drops of PBS/0.5% BSA and washed 3 times. Further, the exosomes were incubated in 5 μl drops of protein A-gold conjugates diluted in blocking buffer for 20 mins followed by 7 washes in PBS. The grids were transferred to 50 μL drops of 1% glutaraldehyde for 5 mins to stabilize immunoreaction. The grids were washed 7 times in 100 μl drops of double distilled water, each time for 2 mins. The samples were contrasted using uranyl oxalate at pH 7 for 5 mins. Immuno-gold labelling was done by Dr. Giulia Mastroianni (TEM Facility Manager) at QMUL, following published protocol [16].

### Dynamic light scattering

Fractions of apoptotic cell debris (1st pellet following 500×g centrifugation), microvesicles (2nd pellet following 16,500×g) and exosomes (3rd pellet following 118,000×g) were resuspended in 1 mL molecular grade water for use in a Zetasizer Nano ZS instrument (Model: ZEN3600, Serial no.: MAL500457, Malvern Instruments) to measure the Brownian motion of particles in a sample using Dynamic Light Scattering providing 3 fundamental parameters of nano-sized particles or molecules in a liquid medium: particle size; zeta potential; and molecular weight. The Zetasizer Nano ZS can measure: particle size for the size range 0.6 nm to 6 μm; Zeta potential for a size range 5 nm to 10 μm; Molecular weight in the size range 1000 to 2 × 10^7^ Da. All samples were read at 4 °C and particle size was measured as intensity percent with respect to diameter in nanometre (nm).

### Nanoparticle tracking analysis

Particle size verification of exosomes was carried out in School of Pharmacy, University College London using NanoSight Nanoparticle Tracking Analysis (NTA NanoSight, Malvern Inc., United Kingdom) [17, 18]. Samples were prepared in 500 μl molecular-grade DNase/RNase/Protease-free water (W4502, Sigma-Aldrich) and stored at − 80 °C until use. Using a 1-mL syringe, samples were loaded into the assembled sample chamber of the NanoSight LM10. One minute video images were captured with manual shutter and gain adjustments (Hamamatsu C11440 ORCA-Flash 2.8 digital camera) and analysed using the NanoSight NTA 2.0 software.

### RNase protection assays and RNA isolation and quality control

RNase, detergent and protease protection assays were performed to investigate the origin of RNA whether it was protected by exosomes and/or protein complexes. Following ultracentrifugation, the supernatant was gently aspirated and the exosome pellet was resuspended in 260 μL molecular grade water and subdivided into equal fractions (50 μL) which received either: 1) vehicle (dH_2_0), 2) RNase A digestion (0.6 mg/mL final concentration, #R6513, SIGMA) (30 min at 37 °C), 3) detergent incubation (2% Triton-X, 10 min at 55 °C) followed by RNase digestion, 4) proteinase K digestion (PK, #03115828001, ~ 0.4 mg/mL final concentration, Roche Diagnostics) (10 min at 55 °C prior to 5 min heat inactivation at 95 °C) followed by RNase digestion, 5) Triton-X and PK treatment followed by RNase digestion. RNase activity was then inactivated by adding RNase inhibitor (03335399001, 1 U/μl final, Roche, 5 mins at RT). Total RNA was purified using RNeasy Micro Kit (#74004, Qiagen) and quantified using Quan-iTTM RiboGreen® RNA Assay kit (R11490, Molecular Probes, Life Technologies). RNA size, quality and relative quantity were assessed by Agilent BioAnalyzer RNA 6000 Pico chip (#5067–1513, Agilent Technologies, Germany). Our typical exosomal RNA yield from each cell line sample (grown in 2× T157 flasks with a total of 80 mL culture supernatant) was 1–4 ng (10–50 pg RNA/mL supernatant). Given that on average each mL of supernatant contains 1-5 × 10^7^ particles (determined by NTA), we estimated each exosome to contain approximately 0.2-5 × 10^−18^g (attogram) RNA.

### Reverse transcription-quantitative PCR (RT-qPCR)

Exosomal RNA were converted to cDNA using qPCRBIO cDNA Synthesis kit (#PB30.11–10, PCRBIO Systems, UK) and the cDNA was diluted 1:4 with RNase/DNase free water and stored at − 20 °C until used for qPCR. Relative quantitative PCR were performed using qPCRBIO SyGreen Blue Mix Lo Rox (#PB20.11–50, PCRBIO Systems, UK) in the 384-well LightCycler 480 qPCR system (Roche) according to our well-established protocols [11, 12, 19, 20] which are MIQE compliant [21]. Briefly, thermocycling begins with 95 °C for 30s prior to 45 cycles of amplification at 95 °C for 6 s, 60 °C for 6 s, 72 °C for 6 s, 76 °C for 1 s (data acquisition). A ‘touch-down’ annealing temperature intervention (66 °C starting temperature with a step-wise reduction of 0.6 °C/cycle; 8 cycles) was introduced prior to the amplification step to maximise primer specificity. Melting analysis (95 °C for 30s, 65 °C for 30s, 65–99 °C at a ramp rate of 0.11 °C/s with a continuous 1 acquisition/°C) was performed at the end of qPCR amplification to validate single product amplification in each well. Relative quantification of mRNA transcripts was calculated based on an objective method using the second derivative maximum algorithm (Roche). All target genes were normalised to two stable reference genes (YAP1 and POLR2A) validated previously to be uninfluenced by disease process [20]. For further verification and comparison, some experiments were performed using Taqman gene expression assays for FOXM1 (Hs1073586_m1), CEP55 (Hs01070181_m1) and ACTB (Hs01060665_g1) using LightCycler® 480 Probes Master (#04707494001, Roche Diagnostics) with a pre-incubation of 50 °C, 2 min and 95 °C, 10 min hot-start followed by 50 cycles of 95 °C, 10s and 60 °C, 60s.

### Western blotting

Primary Antibody used were: Alix(3A9) (1:1000 dilution, mouse monoclonal, mAb#2171, Cell Signalling), CD9 (1:200, rabbit monoclonal, ab92726, Abcam), CD63 (H-193) (1:1000, rabbit polyclonal, sc-15,363, Santa Cruz), CEP55 (1:10000, rabbit monoclonal, ab170414, Abcam), Calnexin (1:1000, rabbit polyclonal, ab22595, Abcam), Glypican 1 (1:500, rabbit polyclonal, ab55971, Abcam), FOXM1 (1:500, rabbit polyclonal, sc-502, Santa Cruz), GAPDH (1:10000, mouse monoclonal, ab8245, Abcam), HSC70(B-6) (1:10000, mouse monoclonal, SC-7298, Santa Cruz). Secondary antibody used were: Goat anti Rabbit IgG (1:1000; AP#132P, Millipore), Goat Anti-Mouse IgG (1:10000, A0168, Sigma). Additional information on immunoblotting methodology can be found in (Additional file 1).

### Microarray gene expression

Normal primary oral keratinocytes (OK113) cells were seeded (1 × 10^5^ per well) in 6-well plates 1 day prior to transfection with exogenous exosomes derived from 3 normal oral keratinocytes (OK113, NK4, NOK368) and 5 malignant (Ca1, CaLH2, SQCC/Y1, SVpgC2a and SVFN8) cell lines. Exosome concentrations were adjusted to 2 × 10^10^ particles/well. Untreated OK113 cells were used as a control. After 48 h of incubation with exosomes in SFM, the cells were washed with 1 × PBS and lysed in lysis buffer (RLT buffer) for total RNA extraction using RNeasy Micro Kit (#74004; Qiagen). Quality and quantity of total RNA was analysed on Nanodrop 1000 spectrophotometer and Agilent BioAnalyzer prior to transcriptome profiling using Illumina genome-wide gene expression Human HT-12 v4.0 Expression BeadChip surveying 47,231 transcripts per sample (performed at Barts and The London Genome Centre, core facility). The data from microarray was analysed on Genome studio version 3 Gene Expression Module. Raw transcriptome data have been deposited at NCBI’s GEO database (GSE89217).

## Results

### Physical characterisation and verification of Exosomal proteins

**Fig. 1:**
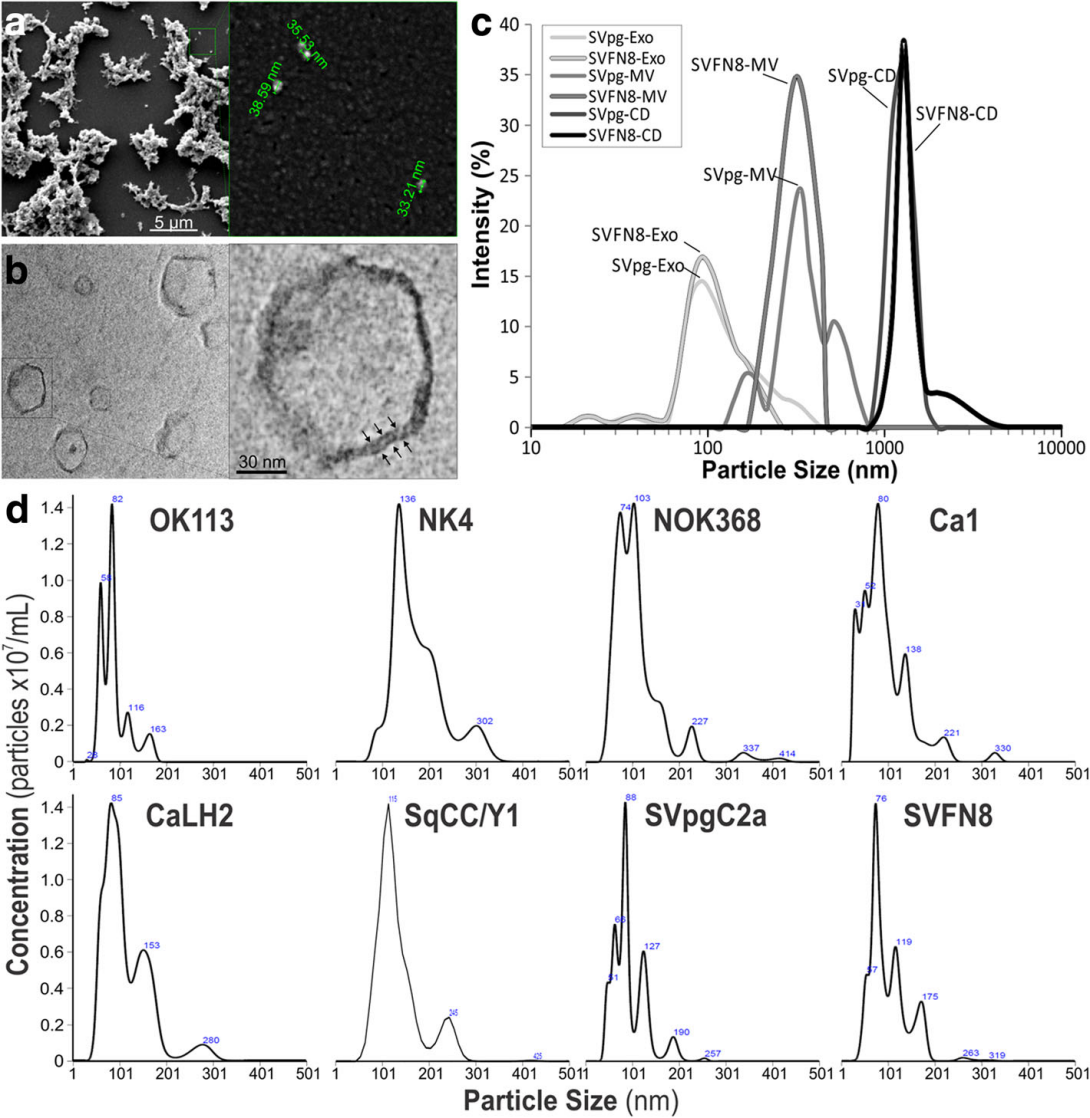
Physical characterisation of exosomal size and concentrations. **a** scanning electron microscopy at low magnification (left panel) and a subset showing high magnification (right panel) showing approximate diameters of each particle. **b** Transmission electron microscopy at low magnification (left panel) and a subset showing high magnification (right panel), note the arrows indicating lipid-bilayer membrane structure. **c** Zetasizer measurements on exosomes (Exo), microvesicles (MV) and cell debris (CD) fractions of two cell lines SVpgC2a and SVFN8. **d** NanoSight particle analysis on exosomes derived from 3 normal primary human oral keratinocytes (OK113, NK4 and NOK368) and 5 malignant (Ca1, CaLH2, SqCC/Y1, SVpgC2a and SVFN8) HNSCC cell lines. Numbers indicated within the diagram indicates the peak size (nm)

To confirm exosomal particle size, shape, membrane structure and particle concentrations, we have used a number of different techniques (Fig. 1) including scanning electron microscopy (SEM; Fig. 1a), transmission electron microscopy (TEM; Fig. 1b), Zetasizer (Fig. 1c) and Nanoparticle Tracking Analysis (NTA; Fig. 1d). SEM showed that exosomal sample appeared in clumps and particle size (~ 30–100 nm) appeared to be on average smaller than those measured by TEM (median ~ 50–150 nm), Zetasizer (median ~ 50–150 nm) and NTA (median 30–200 nm). As SEM were not able to reveal internal structures (Fig. 1a), we used TEM to confirm that exosomes were circular membranous structures (Fig. 1b). The Zetasizer system determines particle size by measuring particle Brownian motion in suspension, using dynamic light scattering for particles sizes from 0.3 nm to 10 μm. For differential ultracentrifugation, cell debris and apoptotic bodies (1–5 μm) are pelleted at 500×g centrifugation. Microvesicles (200–800 nm) are pelleted at 16,500×g and exosomes (30–200 nm) at 118,000×g (Fig. 1c). Finally we used NTA to measure each of the 8 different types of exosomes derived from 3 normal oral keratinocytes (OK113, NK4, NOK368) and 5 malignant (Ca1, CaLH2, SQCC/Y1, SVpgC2a and SVFN8) cell lines. Exosomes from these cell lines showed median sizes ranging from 76 to 136 nm (Fig. 1d) which are consistent with published findings [22]. We did not see any significant physical differences between normal and cancer exosomes. However, we did notice that TEM imaging showed slightly smaller exosome sizes compared to NTA and Zetasizer. This could be due to differences in sample processing where TEM required exosomes to be dried down before imaging.

**Fig. 2:**
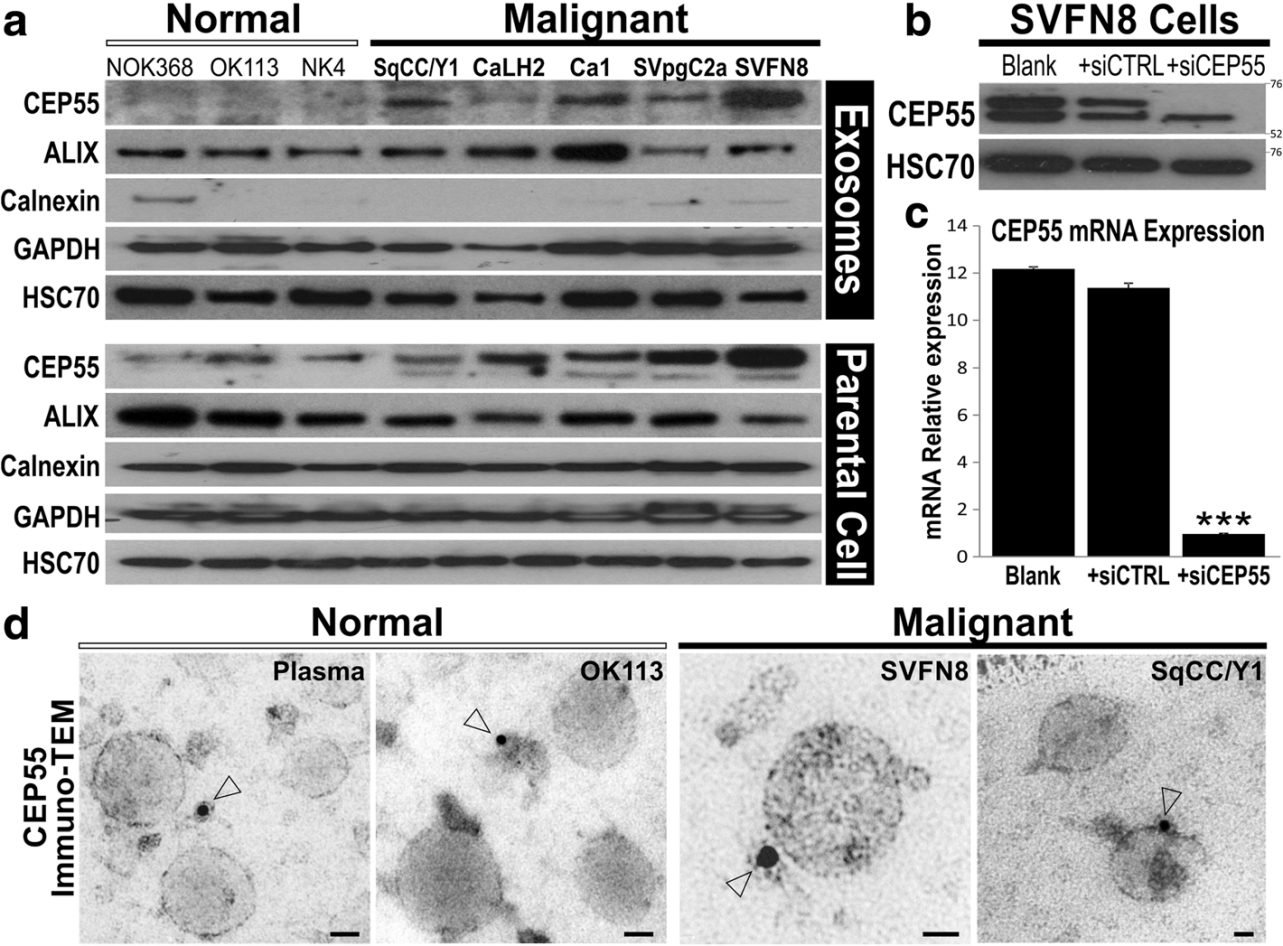
Differential expression of CEP55 in normal and cancer derived exosomes. **a** Immunoblotting for exosomal proteins in normal (NOK368, OK113, NK4) and malignant (SqCC/Y1, CaLH2, Ca1, SVpgC2a, SVFN8) cell-derived exosomes (top panel) and parental cells (bottom panel). ALIX, exosomal protein; Calnexin, endosomal protein; GAPDH and HSC70 were used as loading controls. **b** Verification of CEP55 antibody specificity using siRNA on SVFN8 cell line which expresses high levels of CEP55. HSC70 was used as a loading control. **c** RT-qPCR confirmed that siCEP55, but not siCTRL, significantly (***P < 0.001) knocked down the mRNA of CEP55 in SVFN8. **d** CEP55 protein localisation studying using immunogold-transmission electron microscopy on exosomes derived from normal human plasma, OK113, SVFN8 and SqCC/Y1 as indicated. Open arrow heads indicate CEP55 protein gold labels (< 15 nm diameter including the halo around each black dot). Scale bars represent 30 nm

Purity of exosomes were further verified by immunoblotting for exosomal specific and non-specific proteins. ALIX is an exosomal specific membrane protein and a component of ESCRTiii complex and is involved in the biogenesis of exosomes [5]. It was expressed in exosomes derived from all the eight cell lines at the molecular weight of 102 kDa (Fig. 2a), indicating successful isolation of exosomes. In order to rule out extravesicular protein contamination, calnexin, an endoplasmic reticulum protein aiding in proper protein folding [23], was used to investigate the purity of exosomes. Ideally the expression of calnexin should be absent from exosomes since they lack the endoplasmic reticulum machinery. Calnexin was expressed in all parental whole cell lysates at the molecular weight of 90 kDa, while the expression was very low to absent in the exosome samples, verifying that our exosomes are relatively clean and free from contaminations. Heat shock protein HSC70 (70 kDa) and Glyceraldehyde 3-phosphate dehydrogenase GAPDH (37 kDa) were used as loading controls (Fig. 2a).

Tetraspanin proteins including CD63, CD9 and CD81 have been reported as exosomal specific membrane proteins [24]. In our study we looked for the expression of CD63 (63 kDa) and CD9 (25 kDa) in parental cell lysates and exosomes. We detected CD63 and CD9 proteins in parental cell lysates but in exosomes were almost undetectable despite at maximum protein loading (40 μg/lane) and using highly sensitive detection system (data not shown). Further exploration of the literature indicates that these markers were reported from studies carried out on immune exosomes derived from mast and dendritic cells [25]. Hence, we conclude that there may be variations in these different surface protein markers depending on the origin of parental cell types.

### CEP55 is a potential cancer exosomal membrane marker

We have previously published that CEP55 is a downstream target of FOXM1 oncogene [11, 12] and that CEP55 has been shown to be associated with ALIX and the ESCRT complex [10, 26], we therefore asked if CEP55 protein may be enriched in cancer exosomes as it is known to be upregulated in cancer cells [11, 12, 27]. As expected, the expression of CEP55 protein was found exclusively in exosomes derived from all 5 malignant cell lines and absent from the 3 normal primary oral keratinocytes (Fig. 2a). Within the parental cell lines, CEP55 expressed as doublet at the molecular weight of 55 kDa. In order to verify the specificity of our antibody and exclude non-specific binding, CEP55 was knocked down in SVFN8 cells by siRNA transfection (Fig. 2b). The successful knockdown of CEP55 mRNA in siCEP55 transfected SVFN8 cells was validated by RT-qPCR (Fig. 2c). Silencing of CEP55 protein by siCEP55 led to the disappearance of the top band (Fig. 2b) indicating that the top band was the correct CEP55 protein band. Given that CEP55 protein was found to be enriched in exosomes from cancer cell lines, we further investigated if this protein was specific to cancer exosomes or non-specifically co-purified with exosomes. We therefore performed immuno-gold TEM on exosomes to directly visualise CEP55 protein in exosomes isolated from a normal healthy blood plasma, normal oral keratinocyte cell line (OK113) and two malignant cell lines (SVFN8 and SqCC/Y1; Fig. 2d). Exosomes were incubated with CEP55 antibody and the gold labels were primed against CEP55 antibody. Immuno-TEM images showed that CEP55 gold labels appeared to be clustered by debris (< 15 nm diameter; Fig. 2d) in normal plasma and OK113 exosomes while SVFN8 and SqCC/Y1 exosomes showed CEP55 gold labels appeared on the outer membrane of exosomes. Although not quantitative, these results provided qualitative confirmation that CEP55 could be a specific cancer exosomal membrane marker. Whilst this is beyond the scope of the current study, further validation on clinical specimens are required.

### Verification of RNA cargos of exosomes

**Fig. 3:**
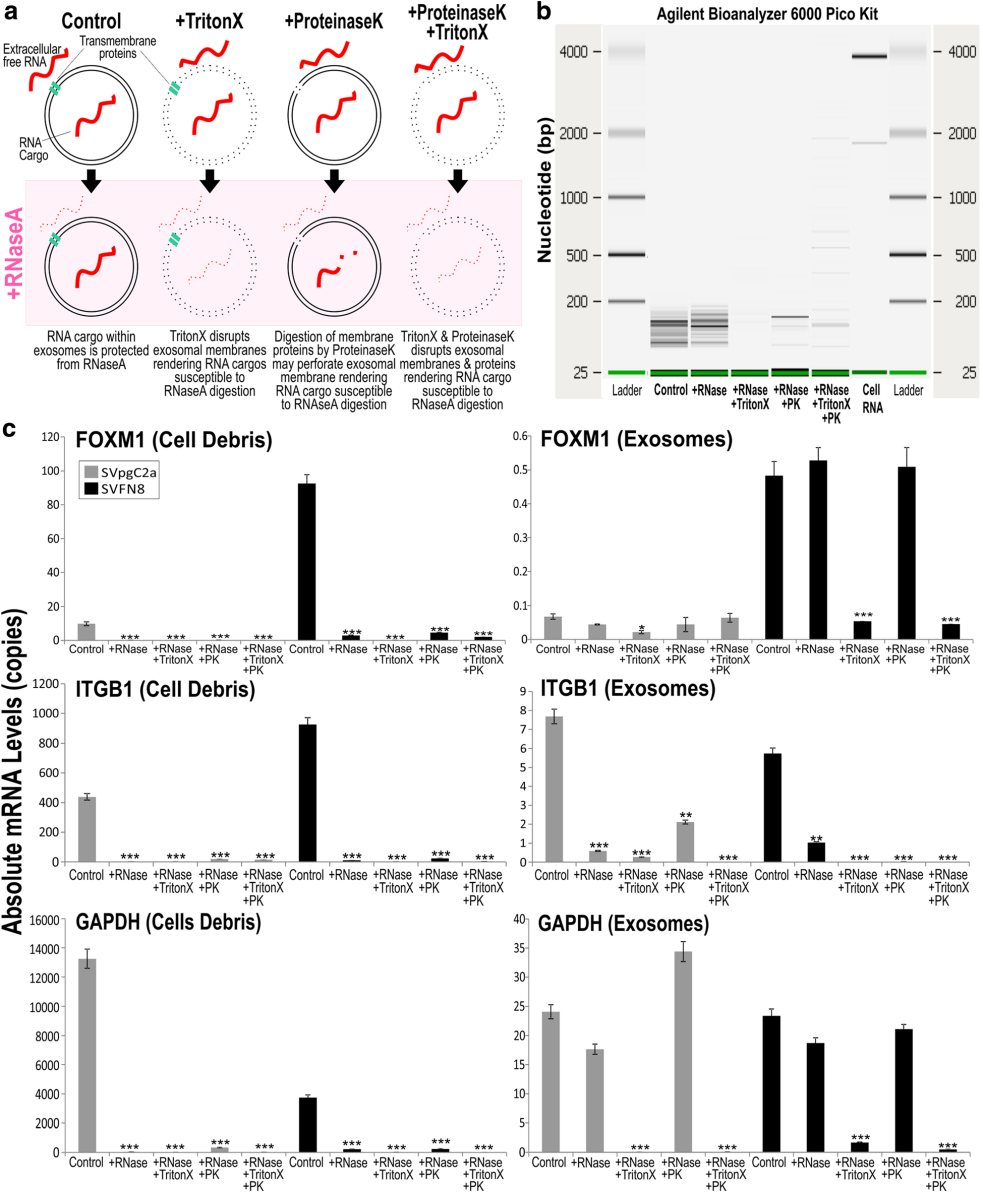
RNA cargo of exosomes is protected from RNase. **a** schematics showing that RNA molecules are protected within intact exosomal membranes which is resistant to RNase activity. TritonX disrupts exosomal membranes and thereby rendering RNA cargos susceptible to RNase digestion. Digestion of membrane ProteinaseK may perforate membrane proteins thereby rendering RNA cargo susceptible to RNase digestion. **b** Exosomal RNA quantification and quality confirmation (using Agilent BioAnalyzer 6000 Pico Kit) showing that majority of exosomal RNA are below 200 bp and are resistant to RNase digestion until addition of TritonX and/or ProteinaseK. **c** RT-qPCR confirmed differential mRNA sorting into exosomes. FOXM1, but not ITGB1, were found to be specifically packaged within exosomes (therefore resistant to RNase digestion). FOXM1 (isoform B) is constitutively overexpressed in SVFN8 cell line. ***P < 0.001 indicates statistically different from control

Free RNA or complexes containing RNA may be co-precipitated alongside exosomes during ultracentrifugation. To confirm that RNA cargos of exosomes are protected against RNase digestion (Fig. 3a), we performed RNase and ProteinaseK protection assays on purified exosomes. Using Agilent BioAnalyzer analysis, we confirmed that exosomal RNA remained intact (< 200 bp) following incubation with RNaseA (Fig. 3b). Addition of TritonX to exosomes disrupted exosomal membranes rendering exosomal RNA susceptible to RNaseA digestion. Interestingly, we also found that addition of ProteinaseK to exosomes also rendered exosomal RNA partially susceptible to RNaseA digestion. We hypothesised that ProteinaseK may digest exosomal membrane proteins thereby perforating exosomal membrane rendering RNA cargo susceptible to RNaseA digestion. Having verified that RNA cargos were indeed protected within exosomes, we asked whether mRNAs were protected and found within exosomes. We have chosen SVpgC2a (a premalignant cell line) and SVFN8 (a malignant cell line derived from SVpgC2a, constitutively expressing FOXM1) [12]. SVpgC2a expresses low levels of FOXM1 oncogene whilst SVFN8 expresses high levels of FOXM1. We compared cell debris and exosomes purified from supernatant of these two cell lines, following the same RNase and ProteinaseK protection assays as shown in Fig. 2b, we performed RT-qPCR to measure the levels of FOXM1, ITGB1 and GAPDH mRNA (Fig. 3c). FOXM1, ITGB1 and GAPDH mRNA purified from cell debris fraction were all susceptible to RNaseA and ProteinaseK, indicating that mRNA were not protected within vesicles. On the other hand, we found that FOXM1 and GAPDH, but not ITGB1, mRNAs were resistant to RNase digestion. We also screened for other oncogenes including FOXM1B, HOXA7, CCNB1, CENPA, DNMT3B, DNMT1, CEP55, NEK2, HELLS, and BMI1. We found low levels of mRNA protected within exosomes treated with RNase. Of these, FOXM1B and HOXA7 mRNA levels were more abundant in SVFN8 exosomes compared to SVpgC2a exosomes. Whereas MAPK8, AURKA and ITGB1 mRNA were degraded with RNase treatment suggesting they were not cargos of exosomes but co-purify with protein aggregates during isolation (Additional file 1: Figure S1). Using different qPCR primers to probe for the same mRNA transcript (EGFP-FOXM1B) in exosomes, RT-qPCR results suggest that EGFP-FOXM1B mRNA transcripts were full length (Additional file 1: Figure S2A). We concluded that exosomal mRNA is protected from extracellular environment including RNase and proteinase. Furthermore, we demonstrated evidence of differential mRNA sorting mechanism whereby some genes were preferentially packaged within exosomes.

### Transcriptome analysis on exosome transfected oral keratinocytes

Having characterised and validated our exosomes, we investigated if these purified exosomes were functionally active and could trigger a response in recipient normal oral keratinocytes. Our preliminary experiments involved transfecting exosomes, derived from a premalignant SVpgC2a and a malignant SVFN8, on SVpgC2a as recipient cells. We found that exosomes from SVFN8 (containing high levels of EGFP-FOXM1B mRNA cargo; Additional file 1: Figure S2A), but not SVpgC2a, triggered an obvious morphological change resembling senescence and/or differentiation within 24 h following transfection in SVpgC2a cells (Additional file 1: Figure S3A). We confirmed the transfer of mRNA cargo (EGFP-FOXM1B) into recipient cells using qRT-PCR to detect EGFP (Additional file 1: Figure S2B) but it was surprisingly low levels and transient (only detectable at 24 and 48 h, but not 72 or 96 h following transfection, data not shown). This led us to investigate if exosome exposure trigger cell senescence and/or differentiation in recipient cells. No evidence of senescence associated β-galactosidase activity nor significant mRNA modulation of senescence/apoptotic genes p53, p21, p16 and CBX7 suggesting that recipient cells were not undergoing senescence following exosome exposure (Additional file 1: Figure S3B). Instead, we found some evidence that mRNA of differentiation markers cornifin (CORN) and loricrin (LORI) were perturbed, but not involucrin (IVL) or transglutaminase 1 (TGM1), in recipient SVpgC2a cells indicating exosomes does not directly activate differentiation but may have some roles in modulating differentiation (Additional file 1: Figure S3C). As these data provided incomplete picture, we therefore opted for transcriptome analysis.

**Fig. 4:**
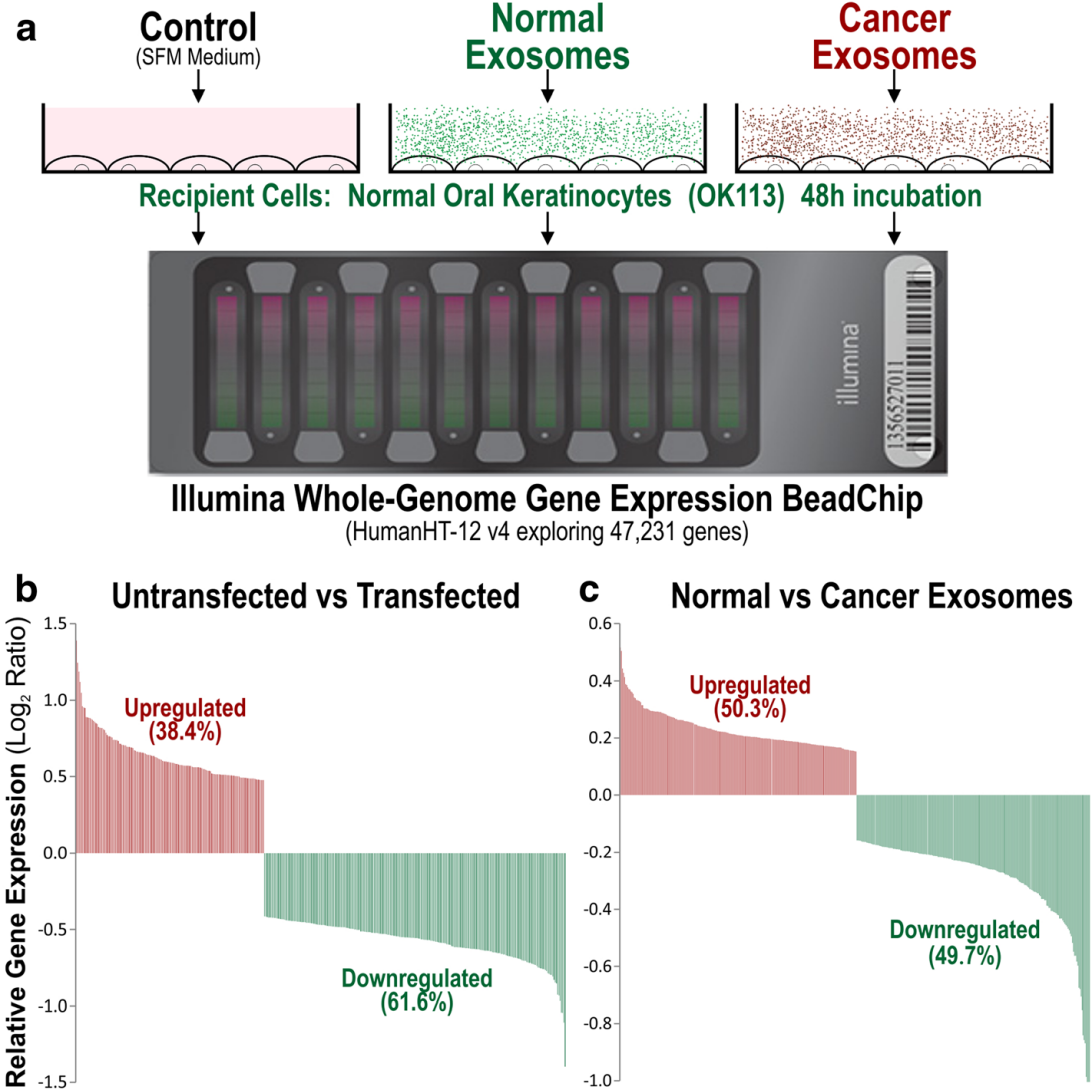
Genome-wide gene expression analysis on normal primary human oral keratinocytes transfected by normal or cancer-derived exosomes. **a** OK113 cells were transfected by equal concentration (2 × 10^10^ particles/well) of each type of exosomes for 48 h prior to microarray analysis using Illumina Human HT-12 v4 Gene Expression BeadChip, exploring 47,231 genes. **b** Within the top 400 differentially expressed genes (*P* < 10^− 20^), exosome (both normal and cancer) exposure led to larger proportion of downregulated genes (61.6%) compared with upregulated genes (38.4%) in recipient cells. **c** Within the top 400 differentially expressed genes (*P* < 10^− 2^), cancer and normal exosomes induced almost equal proportion (50.3 vs 49.7%) of differentially expressed genes in recipient cells

**Fig. 5:**
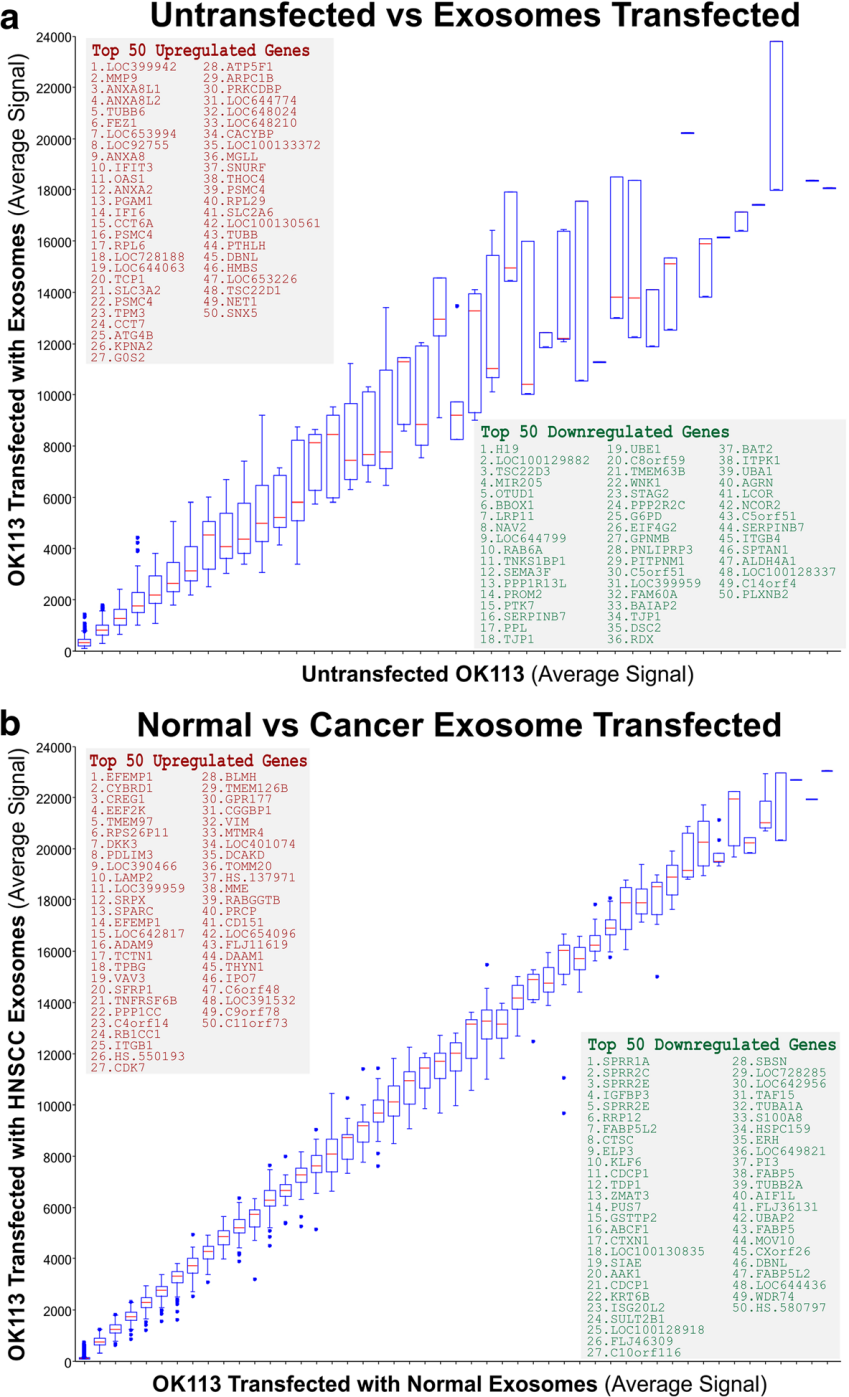
Genome-wide differential gene expression analysis on normal primary human oral keratinocytes transfected by normal or cancer-derived exosomes. **a** Correlation box-whisker plot between untransfected vs exosome (including both normal and cancer exosomes) transfected gene expression profiles. **b** Correlation box-whisker plot between normal vs cancer exosome transfected gene expression profiles. Insets showing top 50 upregulated and top 50 downregulated genes, respectively

To our knowledge at the time of this study, no publication has compared transcriptomes of primary normal human oral keratinocyte cells transfected with normal or cancer exosomes. Using microarray, we investigated the global gene expression profile (47,231 genes) in an unbiased way to understand the functional effects in recipient cells exposed to normal vs cancer exosomes. Exosomes isolated from normal primary human oral keratinocytes (OK113, NK4, NOK368), HNSCC tumour-derived cell lines (Ca1, CaLH2, SqCC/Y1), premalignant buccal oral keratinocytes (SVpgC2a) and transformed malignant cell line (SVFN8), were transfected onto OK113 cells for 48 h in serum free medium prior to harvest. Un-transfected OK113 cells were used as a control. When comparing untransfected cells with all exosome-transfected cells, within the top 400 differentially expressed genes, 61.6% genes were downregulated and 38.4% were upregulated (Fig. 4b). But when comparing between cancer and normal exosome-transfected cells, within the top 400 differentially expressed genes, cancer and normal exosomes induced almost equal proportion (50.3 vs 49.7%) of differentially expressed genes in recipient cells (Fig. 4c). Correlation box-whisker plot between untransfected vs exosome-transfected cells showed significantly larger differential gene expression (Fig. 5a) compared to that between cancer vs normal exosome transfected cells (Fig. 5b), indicating that exposure to either normal or cancer exosomes triggered significant change in the transcriptome of recipient cells. We further studied the differential gene expression between normal vs cancer exosomes-transfected cells and found that cancer exosomes indeed triggered different sets of genes to those triggered by normal exosomes.

**Fig. 6:**
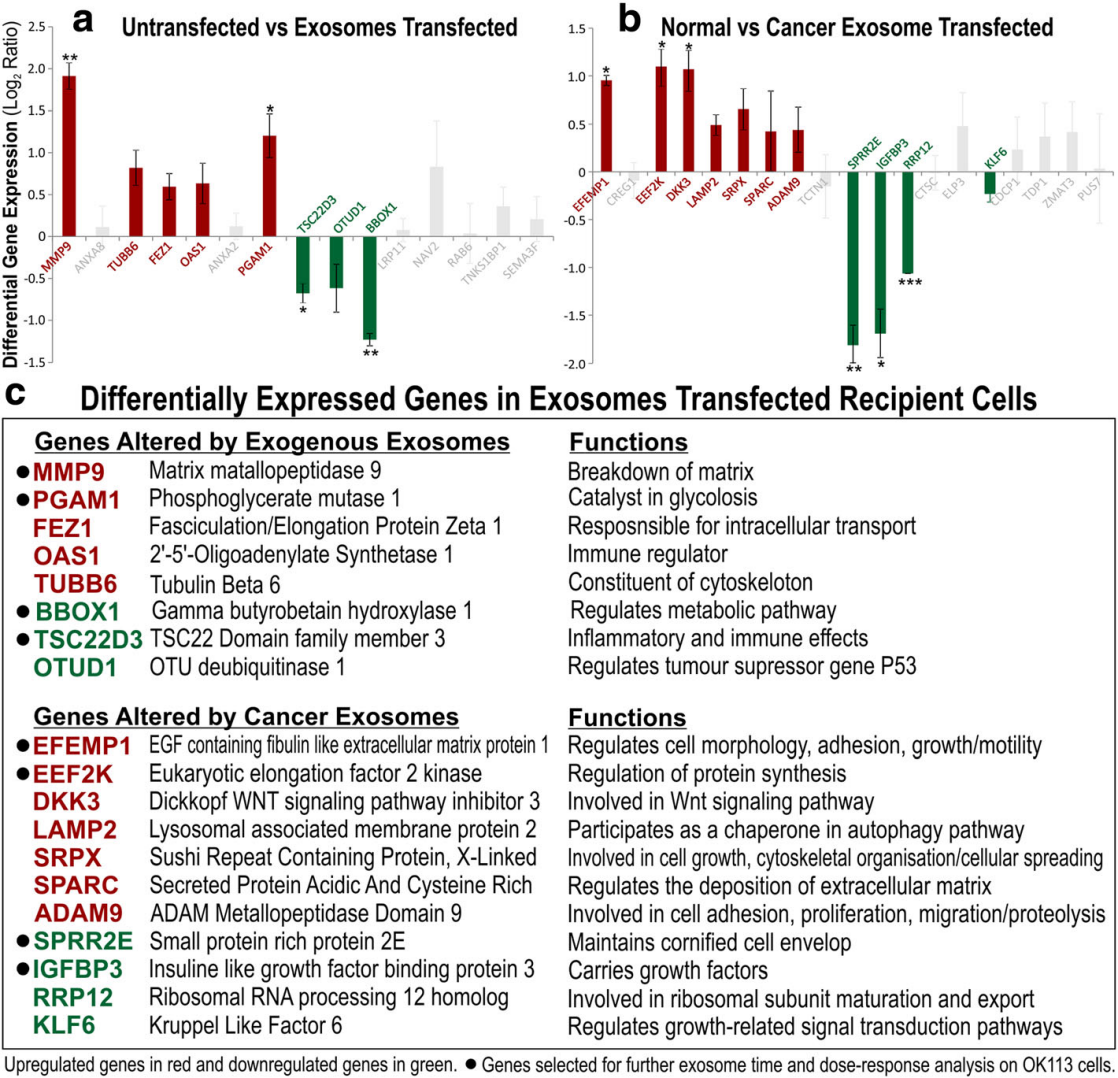
Verification of candidate gene differential expression using RT-qPCR on recipient OK113 cells. **a** Each bar represents differential mean ± SEM gene expression (Log_2_ Ratio) of 8 exosomes (NOK368, OK113, NK4, SqCC/Y1, CaLH2, Ca1, SVpgC2a, SVFN8)-transfected OK113 compared with untransfected OK113. **b** Each bar represents differential mean ± SEM gene expression (Log_2_ Ratio) of 5 cancer exosomes (SqCC/Y1, CaLH2, Ca1, SVpgC2a, SVFN8)-transfected OK113 vs 3 normal exosomes (NOK368, OK113, NK4)-transfected OK113. Coloured bars (red for upregulated and green for downregulated genes) represent differential expression patterns correlated with results obtained by microarray experiments in Fig. 5. Grey bars represent insignificant and/or discordance expression patterns with results obtained by microarray. Statistical t-test *P < 0.05, **P < 0.01 and ***P < 0.001. **c** table listing all validated candidate genes with brief description of their putative functions. Further 8 genes were selected for exosome time and dose-response analysis on OK113 cells in Fig. 7

**Fig. 7:**
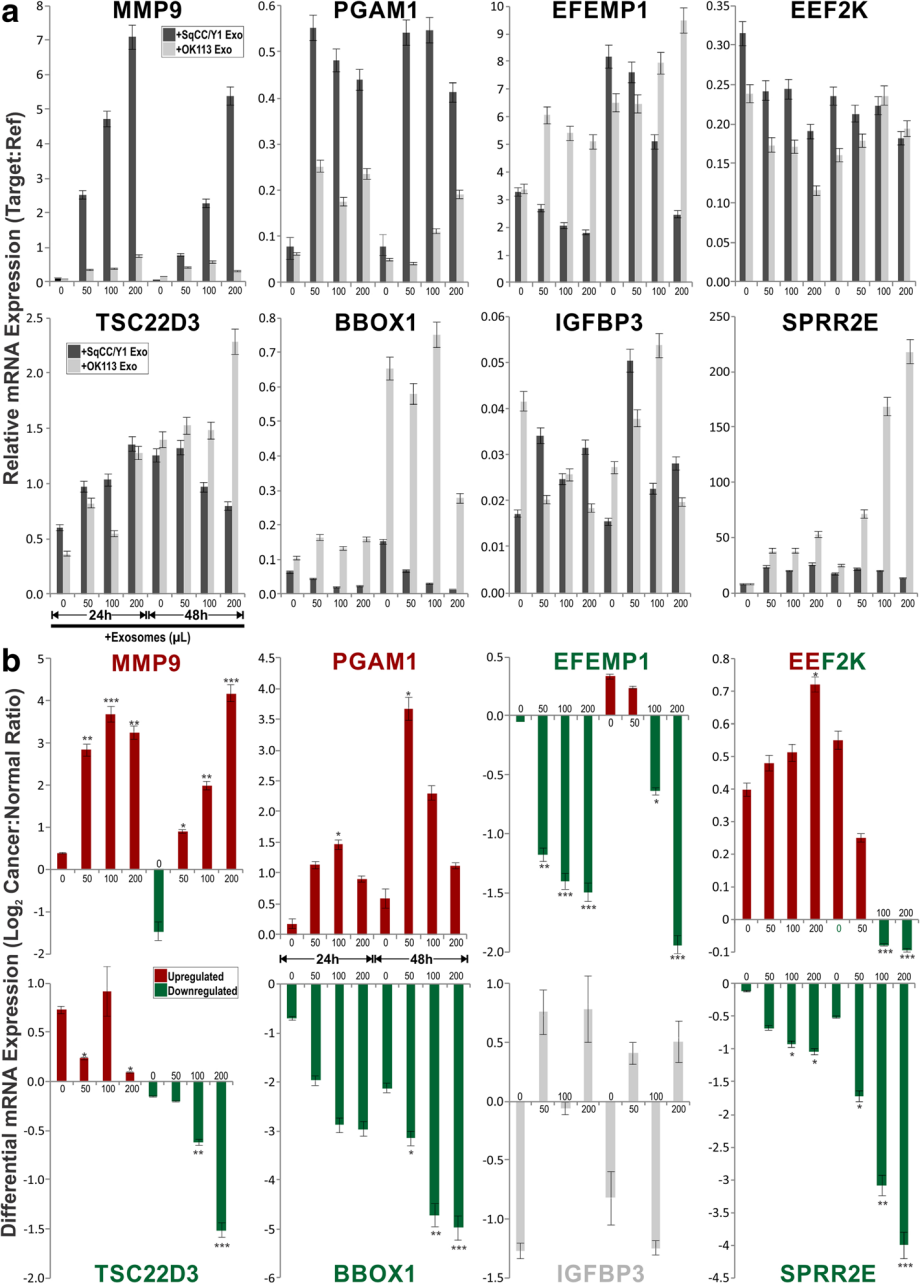
Exosome time and dose-response effects on candidate genes (selected from Fig. 6) analysis on primary normal human oral keratinocytes (OK113). OK113 cells were transfected by different doses (0, 50, 100, 200 μL) of exosomes derived from either OK113 or SqCC/Y1 HNSCC cells. Transfected OK113 cells were harvested at two time points (24 h and 48 h) and RT-qPCR were performed to measure each target gene relative expression. **a** Each bar represents mean ± SEM of relative gene expression (target:reference genes) at each exosome transfection time and dose as indicated. **b** Each bar, derived from data presented in panel **a**, represents mean ± SEM of differential gene expression (Log_2_ Cancer:Normal Ratio) between SqCC/Y1:OK113 exosome transfected OK113 cells at each time and dose of exosomes. Statistical t-test *P < 0.05, **P < 0.01 and ***P < 0.001

Within the top 50 upregulated and downregulated genes in untransfected vs transfected (Fig. 5a) and in cancer vs normal (Fig. 5b), 7–10 genes were selected from each category (total 34 genes) for further validation by RT-qPCR (Fig. 6a, b) using the same RNA samples used for transcriptome analysis. Of the 34 candidate genes, we found that only 19 genes were in agreement with the transcriptome data. Their associated gene functions were listed in Fig. 6c. Of these genes, we further selected 8 genes for exosome dose- and time-response on independent OK113 transfection experiments (using OK113 and SqCC/Y1 derived exosomes) to confirm their functional specificity. We found a mixture of dose and time-dependent gene expression patterns for different genes responding to different exosomes. For MMP9 and PGAM1, both normal (OK113) and cancer (SqCC/Y1) exosomes triggered dose-dependent upregulation of MMP9 and PGAM1, but cancer exosomes were significantly more potent than normal exosomes (Fig. 7a). Conversely, cancer exosomes triggered dose-dependent inhibition of BBOX1 and EFEMP1. Both normal and cancer exosomes activated SPPR2E but cancer exosomes were significantly less potent than normal exosomes (Fig. 7a). Interestingly, cancer exosomes triggered a time-dependent bi-phasic effects on TSC22D3 and EEF2K gene expression whereby at 24 h incubation, they were dose-dependently upregulated but were then downregulated at 48 h incubation with cancer exosomes (Fig. 7a). Neither normal nor cancer (SqCC/Y1) exosomes had any significant effects on IGFBP3 gene expression. Figure 7b summarised the differential mRNA expression ratios of each gene, based on data presented in Fig. 7a.

## Discussion

To our knowledge, this is the first study characterising exosomes secreted from primary human oral keratinocytes and compared with exosomes derived from HNSCC cell lines. Exosomes purified through differential ultracentrifugation from 3 different strains of primary normal human oral keratinocytes and 5 malignant cell lines were subjected to various nanoparticle characterisation methods including scanning electron microscopy, transmission electron microscopy, dynamic light scattering (Zetasizer), 3D Brownian motion (NTA) and biochemical membrane protein and mRNA cargo characterisations. All physical characteristics of our purified exosomes were consistent with published data [18, 22].

During characterisation of exosomal proteins, we accidentally identified a potential unique cancer exosomal membrane protein, CEP55, which were present in all 5 cancer exosomes and absent in all 3 normal exosomes (Fig. 2a). Further analysis using immune-gold TEM confirmed that CEP55 protein was located on the membrane of cancer-derived but not normal exosomes (Fig. 2d). This appears to be consistent with the biosynthesis of endosome involving the ESCRT (endosomal sorting complex required for transport) membrane budding machinery [28]. CEP55 (55 kDa) is a centrosomal protein involved in cytokinesis [29] and a known downstream target of FOXM1 oncogene in HNSCC [30] and breast cancer [31]. Crystal structural study revealed that CEP55 is a partner of ALIX in ESCRT complex [10] involved membrane abscission during viral budding [26] and exosomal budding into multivesicular endosomes [32]. Midbody (Flemming body) typically has a diameter of ~ 1 μm and length of 3–5 μm [33] would have been removed in our exosome purification protocol, hence we could rule out midbody contamination in our exosome preparation. There was also reports demonstrating ESCRT independent mechanism for budding exosomes [32]. The presence of CEP55 on cancer exosomes but not normal exosomes led us to suggest the involvement of different endosome biosynthetic pathways. The mechanism of exosomal budding is beyond the scope of this study, further investigations are required to delineate the role of CEP55 in cancer exosomes.

Consistent with published studies that mRNA cargos are protected within exosomes and resistant to RNase degradation [2], we further showed that digestion of membrane proteins by proteinaseK may render the RNA cargo partially susceptible to RNase degradation presumably because digestion of transmembrane proteins could perforate the membrane of exosomes (Fig, 3a). When screening for candidate mRNA transcripts packaged within cancer exosomes, we found selective cargo loading of certain mRNA transcripts within exosomes, thereby providing evidence of selective sorting of mRNA into exosomes, consistent with existing data that protein and RNA molecules are not randomly loaded into exosomes [34]. Nevertheless, we found evidence that full-length exogenous transcripts were packaged within exosomes derived from a cell line (SVFN8) constitutively overexpressing EGFP-FOXM1B (Additional file 1: Figure S2A) and that the mRNA cargo could be delivered into recipient cells albeit transiently (Additional file 1: Figure S2B), consistent with the finding that mRNA cargos are rapidly degraded upon entry into recipient cells [35]. This has implications in tailored engineering of specific exosomal cargos for cancer therapeutics including self-homing targeted anticancer drug delivery and cancer immunotherapy [36].

The morphological changes observed in recipient oral keratinocytes transfected with cancer exosomes resembled senescence but we could not find evidence of exosome-induced senescence as reported in other cell systems [37]. Instead, we found some evidence of perturbed differentiation markers cornifin (CORN) and loricrin (LORI). As little is known about the functional differences and molecular consequences of normal cells responding to exosomes secreted by normal cells compared to those secreted by cancer cells, we therefore performed transcriptome profiling to investigate the global gene expression profile (47,231 genes) in an unbiased way. Transcriptome data showed that regardless of normal or cancer derived, exosomes altered molecular programmes involved in matrix modulation (MMP9), cytoskeletal remodelling (TUBB6, FEZ1, CCT6A), viral/dsRNA-induced interferon (OAS1, IFI6), anti-inflammatory (TSC22D3), deubiquitin (OTUD1), lipid metabolism and membrane trafficking (BBOX1, LRP11, RAB6A). Interestingly, cancer exosomes, but not normal exosomes, modulated expression of matrix remodelling (EFEMP1, DDK3, SPARC), cell cycle (EEF2K), membrane remodelling (LAMP2, SRPX), differentiation (SPRR2E), apoptosis (CTSC), transcription/translation (KLF6, PUS7). These results indicated that cancer exosomes elicited additional transcriptome programmes compared to normal exosomes. However, both normal and cancer exosomes induced a subset of common pathways in recipient cells, indicating a fundamental mechanism involved when responding to any exosomes.

It has previously been shown that activated T cell exosomes could upregulate MMP9 expression in murine melanoma cells [38] and hepatocellular carcinoma-derived exosomes could increase MMP9 secretion in hepatocytes [39]. Upon further dose- and time-dependent exosome transfection experiments validation by qRT-PCR, we found that cancer exosomes induced stronger upregulation of MMP9 and PGAM1 whilst suppressed BBOX1 and EFEMP1, compared to normal exosomes. Interestingly, TSC22D3 and EFF2K showed biphasic time-dependent effects in respond to cancer exosomes. Our results suggest that although some genes were commonly modulated (eg., MMP9 and PGAM1 were upregulated by both normal and cancer exosomes), the effects were significantly amplified by cancer exosomes in recipient cells. Activation of MMP9 may promote cell migration and invasion, whilst PGAM1 may reroute [40, 41] and/or uncouple glycolytic pathways to promote cell migration [42].

Apart from MMP9, none of the other genes (Fig. 6c) had been previously associated with exosomes. BBOX1 has been shown to be essential for transport of fatty acids across the mitochondrial membrane [43]. Through meta-analysis of microarray data across 13 different types of cancers, BBOX1 has been proposed to have an important role in cancer development [44]. EFEMP1, also known as fibulin 3, is a member of the fibulin family of secreted glycoprotein [45] known to regulate cell morphology, adhesion, growth and motility [46]. Recently, upregulation of EFEMP1 has been found in bladder cancer, correlating with increased tumour invasiveness, while knockdown of EFEMP1 restored the invasive and migratory potential [47]. Another study showed that suppression of EFEMP1 reduced migration, invasion and promote apoptosis in brain cells (glioma) [48]. Studies have also reported that EFEMP1 regulates matrix metalloproteinase (MMPs) and tissue inhibitors of MMPs [49]. Our finding that BBOX1 and EFEMP1 were suppressed by cancer exosomes may indicate activation of a protective mechanism in the recipient cells against potentially harmful cargos in cancer exosomes [50].

SPRR2E is part of the human epidermal differentiation complex on chromosome 1q21 and code for precursor proteins of the cornified cell envelop, a structural characteristic of terminally differentiated keratinocytes [51]. In a study on epidermal squamous cell carcinoma, low expression of SPRR2 was noted in malignant keratinocyte cell lines compared to normal suggesting defective terminal differentiation, a characteristic of carcinogenic transformation [52]. Similar expression of SPRR2 has been observed in neoplastic keratinocytes of the anal track [53]. This is consistent with our finding that cancer exosomes had significantly lower transactivation onSPRR2E expression when compared to normal exosomes, suggesting a role of cancer exosomes in antagonising differentiation in recipient cells.

TSC22D3, also known as glucocorticoid-induced leucine zipper (GILZ), is a potent anti-inflammatory protein and plays a role in cell survival [54]. Previous studies have reported increased expression of EEF2K in breast cancer [55] and glioma [56], where it plays a critical role in cell cycle, autophagy and apoptosis [57] making it a potential target for cancer therapy. We found that both TSC22D3 and EEF2K exhibited biphasic dose- and time-dependent expression following cancer exosomes transfection. Given their roles in regulating inflammation and apoptosis, their expression patterns in recipient cells may indicate a complex dynamic network of interacting signals responding to cancer exosomes.

## Conclusion

This study provided evidence that both normal and cancer exosomes modulated unique gene expression pathways in normal recipient cells. Cancer cells may exploit exosomes to confer transcriptome reprogramming in recipient cells that leads to cancer-associated pathologies such as angiogenesis, immune evasion/modulation, cell fate alteration and metastasis. In addition, we also demonstrated that malignant-cell derived exosomes may express surrogate oncogenic markers such as CEP55 membrane protein and carry FOXM1 mRNA cargos. Based on our findings but requires further validation study, we speculate that exosomal CEP55 protein in saliva or blood could be exploited as a cancer biomarker for non-invasive mode of diagnosis and prognosis of HNSCC. In addition, molecular pathways and biomarkers identified from our transcriptomics study may also be clinically exploitable for developing novel liquid-biopsy based diagnostics and immunotherapies.

## Additional file

Additional file 1: **Figure S1.** RT-qPCR confirmed differential mRNA sorting into exosomes. **Figure S2.** Full length mRNA transcripts were found in exosomes and could be transferred into recipient cells. **Figure S3.** Exosomes induce morphological change within 24 h in SVpgC2a recipient cells. (DOCX 981 kb)

## Abbreviations

ALIX: Apoptosis linked gene 2-interacting protein X
cDNA: Complementary DNA
CEP55: Centrosomal protein 55
CORN: Cornifin
DMEM: Dulbecco’s Modified Eagle Medium
EABR: ESCRT and ALIX-binding region
EGFP: Enhanced green fluorescence protein
ESCRT: Endosomal sorting complex required for transport
FBS: Foetal bovine serum
FOXM1B: Forkhead box M1 isoform B
HNOK: Human normal oral keratinocytes
HNSCC: Head and neck squamous cell carcinoma
ILV: Intraluminal vesicle
IVL: Involucrin
LORI: Loricrin
NTA: Nanoparticle tracking analysis
QMUL: Queen Mary University of London
RT-qPCR: Reverse transcription quantitative polymerase chain reaction
SCC: Squamous cell carcinoma
SEM: Scanning electron microscopy
SFM: Serum free medium
TEM: Transmission electron microscopy
TGM1: Transglutaminase 1
VPS4: Vacuolar protein sorting- associated protein 4
VTA1: Vesicle trafficking 1
