# Supplementary material for: Transcriptome reprogramming by cancer exosomes: identification of novel molecular targets in matrix and immune modulation
Source: Mol Cancer. 2018 Jul 16;17:97. doi: 10.1186/s12943-018-0846-5 (PMC6047127; doi:10.1186/s12943-018-0846-5)
Supplement: Supplementary file 1 — Figure S1. RT-qPCR confirmed differential mRNA sorting into exosomes. Figure S2. Full length mRNA transcripts were found in exosomes and could be transferred into recipient cells. Figure S3. Exosomes induce morphological change within 24 h in SVpgC2a recipient cells. (DOCX 981 kb) [file 12943_2018_846_MOESM1_ESM.docx]

# Supporting Information

**Transcriptome Reprogramming by Cancer Exosomes: Identification of Novel Molecular Targets in Matrix and Immune Modulation**

Fatima Qadir^1^; Mohammad Arshad Aziz^1^; Chrisdina Puspita Sari^1^; Hong Ma^2^, Haiyan Dai^2^, Xun Wang^2^; Dhiresh Raithatha^1^; Lucas Girotto Lagreca Da Silva^1^; Muhammad Hussain^1^; Seyedeh P. Poorkasreiy^1^; Iain L. Hutchison^3^; Ahmad Waseem^1^ and Muy-Teck Teh^1,2,41^

^1^Centre for Oral Immunobiology & Regenerative Medicine, Institute of Dentistry, Barts & The London School of Medicine and Dentistry, Queen Mary University of London, England, United Kingdom.

^2^China-British Joint Molecular Head and Neck Cancer Research Laboratory, Department of Oral & Maxillofacial Surgery, Affiliated Hospital & School of Stomatology, Guizhou Medical University, Guizhou, China.

^3^Department of Oral & Maxillofacial Surgery, Barts & The London NHS Trust, London, England, United Kingdom.

^4^Cancer Research Institute, Affiliated Cancer Hospital & Institute of Guangzhou Medical University, Guangzhou, China.

**Methods and Materials**

Cell Culture

Primary normal human oral keratinocytes (OK113, NOK368 and NK4) were extracted from normal oral mucosa tissue donated by disease-free individuals undergoing wisdom tooth extraction (kindly provided by Prof Daniela Costea, University of Bergen, Norway). Normal epidermal keratinocyte cell line N/TERT was derived from cells cultured from specimens of normal human epidermis (strain N). They have been immortalized by retroviral transduction of h-TERT, a telomerase catalytic subunit [1]. HNSCC derived cell lines Ca1, CaLH2, SqCC/Y1 were previously authenticated and have been used in multiple studies in our lab to represent disease state [2, 3]. SVpgC2a is a Simian virus 40 T-antigen-immortalised human buccal keratinocyte cell line, which retains a non-tumourogenic phenotype and can be used as a model of premalignant oral epithelium [4]. They express low levels of FOXM1 oncogene [2, 3]. SVFN8 cell line has been transformed from SVpgC2a by nicotine exposure and FOXM1 retroviral transduction (as a fusion EGFP-FOXM1B gene) hence overexpressing high levels of FOXM1 oncogene [2].

Western blotting

Samples were lysed in sample buffer (4% (w/v) SDS, 20% (v/v) glycerol and 0.125 M Tris-HCL at pH 6.8). Samples were heated at 95ᵒC for 5 mins to denature the protein. Total protein concentration was measured using a BSA standard and the DCᵀᴹ Protein Assay (Bio-Rad, Hamel Hampstead, UK). The total protein was analysed using 10% (v/v) 2-merceptoethanol and 0.004% (w/v) bromophenol blue to the lysate and separated by SDS-PAGE (NuPage Novex 10% bis tris protein gels, 1.0mm, 10 wells). For both gel electrophoresis and the transfer the Novex mini cell system was used (Invitrogen, Paisley, UK). The membranes were blocked with 5% (w/v) fat-free milk for 30 mins and washed for 3x for 10 mins each with TBS-T (20 mM Trizma base, 150 mM NaCl, 1% (v/v) Tween 20). Membranes were than probed with primary antibody (diluted in blocking buffer) overnight at 4°C and washed with TBS-T to prepare for incubation with secondary antibody (diluted in blocking buffer) for 1 hour at room temperature. Membranes were then washed again with TBS-T. Proteins were detected with Amersham ECL prime western blotting detection reagent (GE healthcare life sciences, Little Chalfont, UK). Peroxidase activity was measured in the dark room with autoradiography film (Amersham Hyperfilm ELC, GE Healthcare, Little Chalfont Bucks, UK).


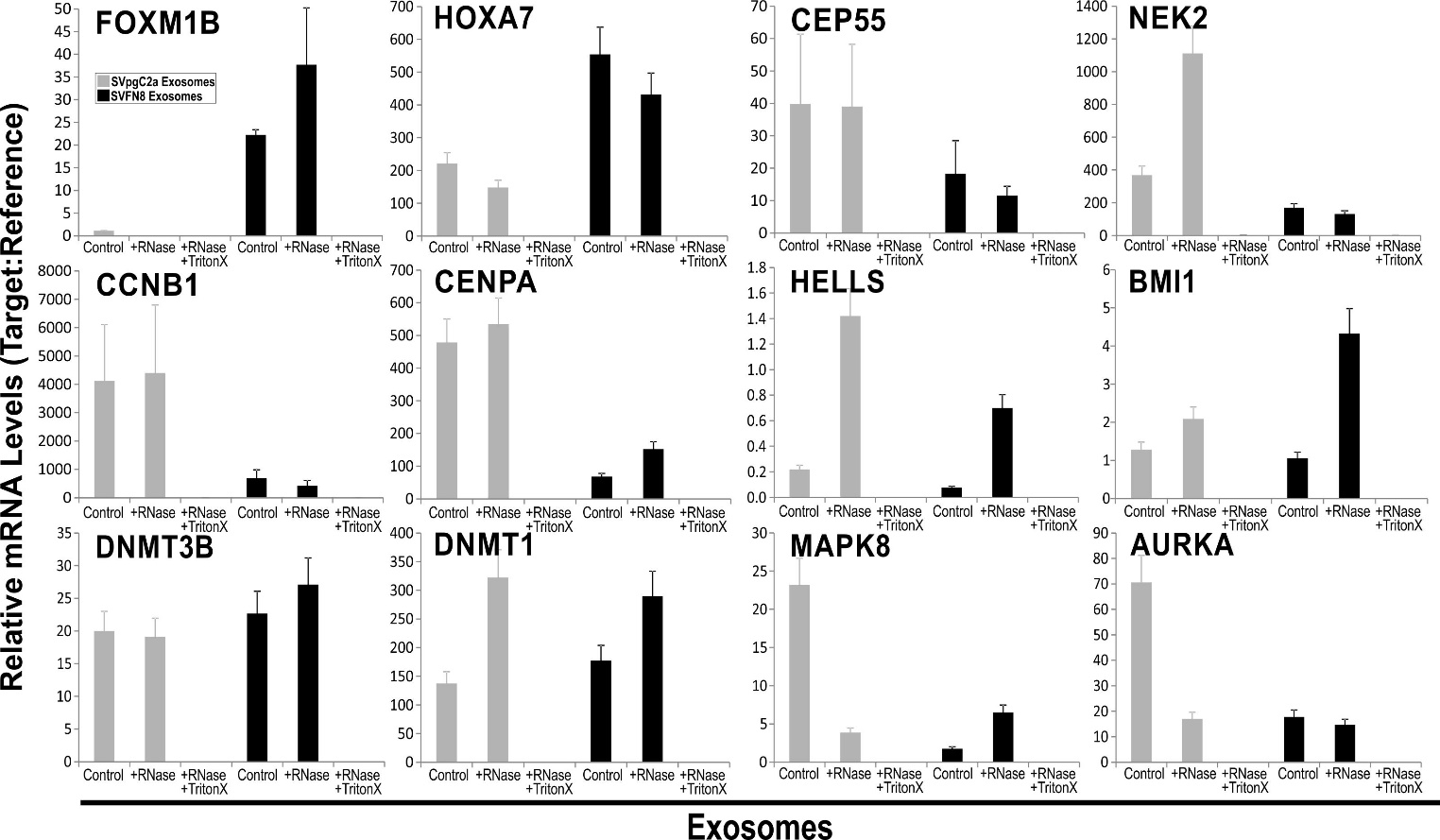


Figure S1 - RT-qPCR confirmed differential mRNA sorting into exosomes. Exosomes purified from SVpgC2a and SVFN8 were subjected to RNaseA or TritonX (or both) treatments prior to RT-qPCR (SYBR Green method) for specific genes as indicated in each panel. Each bar represents the mean ± SEM of quadruplicate determinations. All RNaseA + TritonX treatments led to complete RNA degradation (t-test P>0.001 for all RNaseA + TritonX vs RNaseA treated exosomes for all genes).


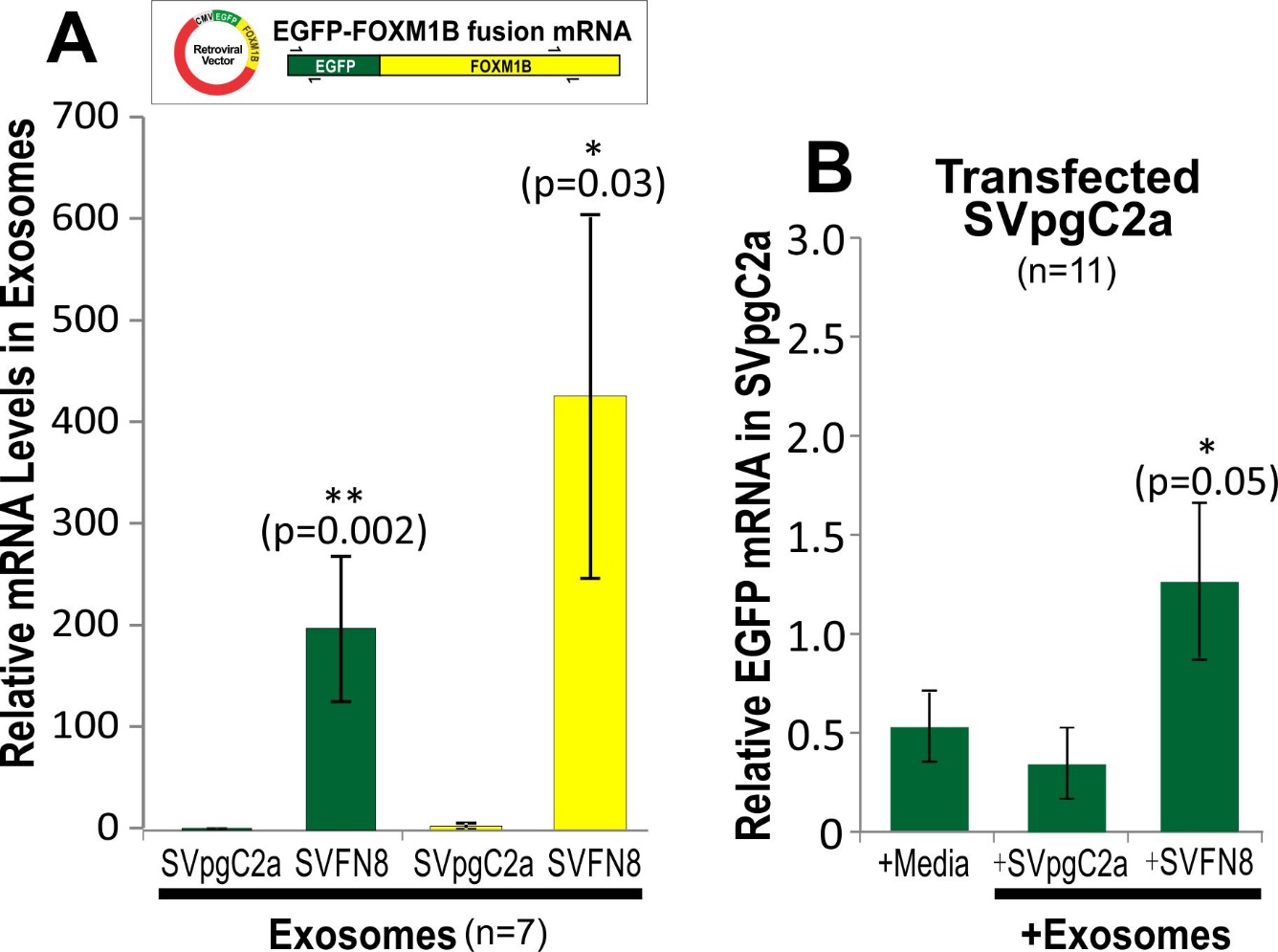


Figure S2 - Full length mRNA transcripts were found in exosomes and could be transferred into recipient cells. A, Using two sets of qPCR primers, one binding against the 3'-terminal of EGFP and the other binding against the 5'-terminal of FOXM1B transcript (as indicated in the diagram above the charts), RT-qPCR were performed on exosomes derived from either control cell line SVpgC2a (not transduced) or SVFN8 cell line constitutively expressing exogenous EGFP-FOXM1B transcripts (transduced with the retroviral vector expressing EGFP-FOXM1B). Expression levels of both EGFP and FOXM1B were highly comparable indicating that the transcript EGFP-FOXM1B were intact. Exosomes derived from SVFN8 contained significantly higher levels of both EGFP and FOXM1B transcripts. B, SVpgC2a cells were either untransfected (+Media) or transfected with exosomes derived from SVpgC2a or SVFN8 for 24h prior to harvest for qRT-PCR to detect the presence of EGFP. Each bar represents a mean ± SEM of n=7 (for experiment in panel A) and n=11 (for B) independent experiments each performed with duplicate determinations. Statistical t-test *P<0.05, **P<0.01.


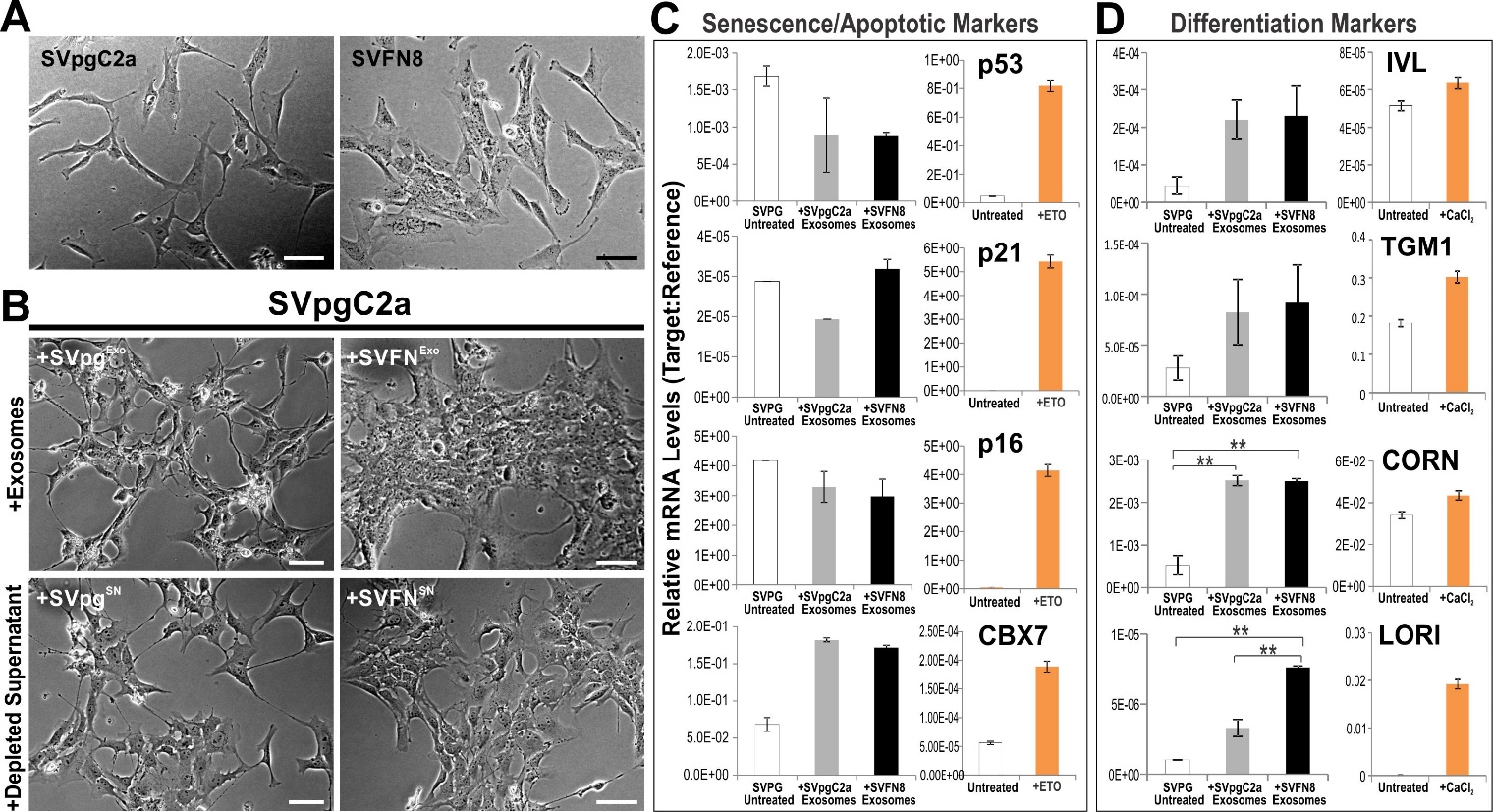


Figure S3 - Exosomes induce morphological change within 24hrs in SVpgC2a recipient cells. A, parental cell morphology, B, transfected SVpgC2a cells after 24hr of incubation with either SVpgC2a-derived exosomes or SVFN8-derived exosomes. Controls were treated with exosome-depleted supernatant, respectively as indicated. Scale bar indicates 30µm. C, Senescence/apoptotic marker genes mRNA were measured in untreated (open bars), SVpgC2a-exosomes (grey bars) or SVFN8-exosomes (black bars) transfected recipient cells (SVpgC2a). Orange bars indicate positive controls treated with etoposide (1x10^-7^ M for 24 hours) to induce senescence. D, Differentiation marker genes mRNA were measured in untreated, SVpgC2a-exosomes or SVFN8-exosomes transfected recipient cells (SVpgC2a). Orange bars indicate positive controls treated with CaCl_2_ (1 mM for 48 hours) to induce differentiation. **P<0.01.

**References**

1. Dickson MA, Hahn WC, Ino Y, Ronfard V, Wu JY, Weinberg RA, Louis DN, Li FP, Rheinwald JG: **Human keratinocytes that express hTERT and also bypass a p16(INK4a)-enforced mechanism that limits life span become immortal yet retain normal growth and differentiation characteristics.** *Mol Cell Biol* 2000, **20:**1436-1447.

2. Gemenetzidis E, Bose A, Riaz AM, Chaplin T, Young BD, Ali M, Sugden D, Thurlow JK, Cheong SC, Teo SH, et al: **FOXM1 upregulation is an early event in human squamous cell carcinoma and it is enhanced by nicotine during malignant transformation.** *PLoS One* 2009, **4:**e4849.

3. Teh MT, Gemenetzidis E, Chaplin T, Young BD, Philpott MP: **Upregulation of FOXM1 induces genomic instability in human epidermal keratinocytes.** *Mol Cancer* 2010, **9:**45.

4. Staab CA, Vondracek M, Custodio H, Johansson K, Nilsson JA, Morgan P, Hoog JO, Cotgreave I, Grafstrom RC: **Modelling of normal and premalignant oral tissue by using the immortalised cell line, SVpgC2a: a review of the value of the model.** *Altern Lab Anim* 2004, **32:**401-405.
